# Muscle Metabolome Profiles in Woody Breast-(un)Affected Broilers: Effects of Quantum Blue Phytase-Enriched Diet

**DOI:** 10.3389/fvets.2020.00458

**Published:** 2020-08-04

**Authors:** Elizabeth Greene, Reagan Cauble, Ahmed E. Dhamad, Michael T. Kidd, Byungwhi Kong, Sara M. Howard, Hector F. Castro, Shawn R. Campagna, Mike Bedford, Sami Dridi

**Affiliations:** ^1^Center of Excellence for Poultry Science, University of Arkansas, Fayetteville, AR, United States; ^2^Department of Animal Sciences, University of Arkansas, Fayetteville, AR, United States; ^3^Biological and Small Molecule Mass Spectrometry Core, Department of Chemistry, University of Tennessee, Knoxville, Knoxville, TN, United States; ^4^AB Vista, Marlborough, United Kingdom

**Keywords:** woody breast, metabolomics, broilers, quantum blue, IPA

## Abstract

Woody breast (WB) myopathy is significantly impacting modern broilers and is imposing a huge economic burden on the poultry industry worldwide. Yet, its etiology is not fully defined. In a previous study, we have shown that hypoxia and the activation of its upstream mediators (AKT/PI3K/mTOR) played a key role in WB myopathy, and supplementation of quantum blue (QB) can help to reduce WB severity via modulation of hypoxia-related pathways. To gain further insights, we undertook here a metabolomics approach to identify key metabolite signatures and outline their most enriched biological functions. Ultra performance liquid chromatography coupled with high resolution mass spectrometry (UPLC–HRMS) identified a total of 108 known metabolites. Of these, mean intensity differences at *P* < 0.05 were found in 60 metabolites with 42 higher and 18 lower in WB-affected compared to unaffected muscles. Multivariate analysis and Partial Least Squares Discriminant analysis (PLS-DA) scores plot displayed different clusters when comparing metabolites profile from affected and unaffected tissues and from moderate (MOD) and severe (SEV) WB muscles indicating that unique metabolite profiles are present for the WB-affected and unaffected muscles. To gain biologically related molecule networks, a stringent pathway analyses was conducted using IPA knowledge-base. The top 10 canonical pathways generated, using a fold-change −1.5 and 1.5 cutoff, with the 50 differentially abundant-metabolites were purine nucleotide degradation and *de novo* biosynthesis, sirtuin signaling pathway, citrulline-nitric oxide cycle, salvage pathways of pyrimidine DNA, IL-1 signaling, iNOS, Angiogenesis, PI3K/AKT signaling, and oxidative phosphorylation. The top altered bio-functions in term of molecular and cellular functions in WB-affected tissues included cellular development, cellular growth and proliferation, cellular death and survival, small molecular biochemistry, inflammatory response, free radical scavenging, cell signaling and cell-to-cell interaction, cell cycles, and lipid, carbohydrate, amino acid, and nucleic acid metabolisms. The top disorder functions identified were organismal injury and abnormalities, cancer, skeletal and muscular disorders, connective tissue disorders, and inflammatory diseases. Breast tissues from birds fed with high dose (2,000 FTU) of QB phytase exhibited 22 metabolites with significantly different levels compared to the control group with a clear cluster using PLS-DA analysis. Of these 22 metabolites, 9 were differentially abundant between WB-affected and unaffected muscles. Taken together, this study determined many metabolic signatures and disordered pathways, which could be regarded as new routes for discovering potential mechanisms of WB myopathy.

## Introduction

Broiler (meat-type) chickens play a key role in worldwide meat production and support the livelihoods and food security of billions of people (1). In fact, poultry meat is highly regarded worldwide as one of the most efficient food sources with high nutrient and organoleptic quality, inexpensive, and without religious taboos (2). However, the emerging woody breast (WB) myopathy is significantly impacting modern broilers and is imposing a huge economic burden on the poultry industry worldwide due to on-farm culling and mortality, down-grading and condemnation at processing, as well as rejection from human consumption (3–7).

WB incidence has increased drastically from an average of 5% in 2012 to 29% in 2015 and has been reported to affect up to 50% in other flocks (8). WB myopathy is emerging on a global scale, already present in Finland (9), France (10), Italy (5), Spain (11), Brazil (12, 13), United Kingdom (14), Japan (15), and in several other countries, and it is negatively impacting global chicken meat production and quality.

Although the etiology of WB is still not well-defined, evidence indicated multifocal degeneration and necrosis of muscle tissue with infiltration of inflammatory cells (9, 16). Lesions associated with the myopathy appear to be aseptic, superficially-located, and include muscle fiber fragmentation, hyalinization, and swelling with replacement by fibrous connective tissue, as well as an influx of macrophages and lipid infiltration (9, 17). These clinical and microscopic changes result in palpable severe hardness of the breast muscle. At molecular and cellular levels, several recent omics studies including transcriptomics (18, 19), metabolomics (8, 20), and proteomics (21) have been conducted and identified numerous potential contributing factors. Of particular interest, hypoxia, oxidative stress, fiber-type switching, cellular damage, and altered intracellular calcium were predicted to play a key role in WB myopathy. In 2016, Abasht's group identified several key metabolites in 48d-old WB-affected purebred lines and commercial broilers (8). In a more recent study using 38d-old Ross 708 broilers and NMR technique, Wang et al. reported alteration of several metabolites including amino acids in WB-affected breasts (20).

In attempt to identify a mechanism-based strategy to reduce the severity of WB, we recently demonstrated that systemic and local hypoxia along with an activation of HIF-1α and its upstream mediators PI3K/AKT/mTOR pathways are responsible for the development of WB myopathy in 56d-old Cobb500 broilers (22). Phytase (quantum blue, QB)-enriched diets at 2,000 FTU/kg reduced the severity of WB via modulation of hypoxia- and oxygen homeostasis-related pathway (22). In the present study, we aimed to gain further insights in the WB etiology and the mode of QB action by using a mass spectrometric metabolomics approach.

## Materials and Methods

### Ethics Statements

The present study was conducted in accordance with the recommendations in the guide for the care and use of laboratory animals of the National Institutes of Health, and the protocol was approved by the University of Arkansas Animal Care and Use Committee (#16084).

### Birds and Diets

The experimental design was previously described (22). Briefly, male broilers (Cobb 500, *n* = 576) were weighed at day one of hatch and randomly assigned to 6 body-weight matched groups in 48 floor pens (12 birds/pen, 8 pens/group) in an environmentally controlled house with *ad libitum* access to feed and water. Birds were fed, for 56 days, one of six dietary treatments in a complete randomized design. The diets were a nutrient adequate positive control (PC) diet formulated to meet Cobb 500 nutrition requirements. *Myo*-inositol (MI, Sigma-Aldrich, St. Louis, MO) was added to the PC diet at 0.30% to create a second diet (PC + MI). The third one was considered the negative control (NC) diet with a reduction of available phosphorus (avP), calcium and sodium by 0.15, 0.16, or 0.03%, respectively. The NC diet was then supplemented with 500, 1,000, or 2,000 phytase units (FTU)/kg to create diets four (NC+500FTU), five (NC+1,000 FTU) and six (NC+2,000 FTU), respectively. The phytase was QB (AB Vista, Marlborough, UK) with an expected activity of 5,000 FTU/g. The diet composition was reported previously (22).

### WB Scoring

Birds (*n* = 85–90/group) were processed at d56 using a commercial inline system at the University of Arkansas Pilot Processing Plant (UAPPP). Breast filets were blind analyzed and macroscopically scored by one trained person (who did not know the different treatments), and classified to WB categories to the degree: 0, normal (NORM); 0.5–1.5, moderate (MOD) with mild hardening in the cranial area; and 2–3, severe (SEV) with severe hardening and hemorrhagic lesions in the cranial region.

### Sample Collection and Preparation

**Figure 1:**
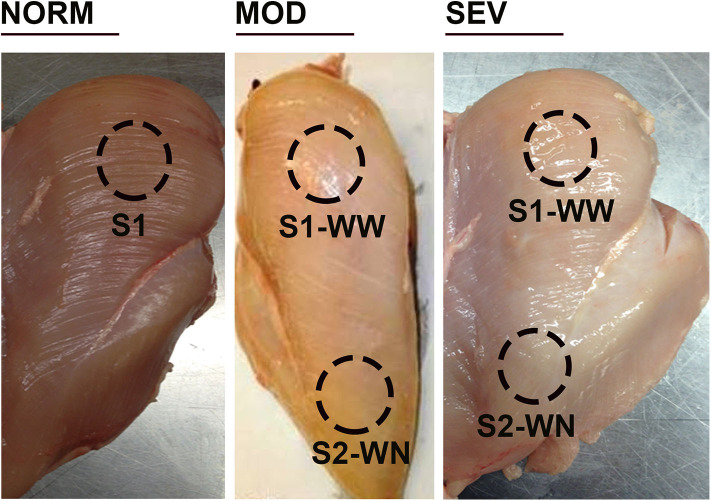
Woody breast categories and sampling locations. Birds were processed at d56, and breast filets were macroscopically scored. NORM, normal breast without harden area; MOD, moderate woody breast with a score between 0.5 and 1.5; SEV, severe woody breast with a score between 2 and 3. S1, cranial surface; S2, caudal surface; WN, woody normal; WW, woody woody.

Breast muscle samples were collected from cranial surface (S1) of unaffected birds and from cranial S1 (woody woody, WW) and caudal S2 (woody normal WN, apparent healthy area) area of WB-affected birds for both MOD and SEV category and from each treatment (*n* = 8, 96 WB-affected with 48 MOD and 48 SEV, and 48 non-affected birds) (Figure 1). Tissues were ground snap frozen in liquid nitrogen, and stored at −80°C until further analysis. Metabolites were extracted with 1.5 mL of extraction solvent (40:40:20 HPLC grade methanol: acetonitrile: water with formic acid at a final concentration of 0.1 M) pre-chilled at 4°C, and incubated at −20°C for 20 min. Samples were centrifuged (13,300 g, 5 min, 4°C) and supernatants were collected. Solvent was evaporated under a stream of nitrogen and metabolites were suspended with 300 μL of ultrapure water.

### Ultra-High Performance Liquid Chromatography—High Resolution Mass Spectrometry (UHPLC–HRMS) Metabolomic Analysis

UHPLC-HRMS analysis has been described previously (23). Briefly, metabolites were separated on a Dionex UltiMate 3000RS (Sunnyvale, CA) by injecting a 10 μL sample on a Synergy reverse phase Hydro-RP 100 Å, 100 mm × 2.00 mm, 2.5 μm pore size LC column (Phenomenex, Torrance, CA) kept at 25°C. The untargeted metabolomics method, adapted from (24), ran for 26 min with the application of multistep gradient. Two HPLC grade solvents were used in gradient steps to separate the analytes. Solvent A contained 97:3 H_2_O: MeOH with 11 mM tributylamine and 15 mM acetic acid and solvent B was 100% MeOH. The gradient was ran as follows: 0 min, 0% B; 5 min, 20% B; 13 min, 55% B; 15.5 min, 95% B; 19 min, 0% B; 25 min, 0% B with a flow rate of 200 μL/min. The eluent was introduced into the mass spectrometer via an electrospray ionization (ESI) source conjoined to an Exactive™ Plus Orbitrap Mass Spectrometer (Thermo Scientific, Waltham, MA) under established parameters of aux gas: 8; sheath gas: 25; sweep gas: 3; spray voltage: 3.00 kV; and capillary temperature: 300°C. The mass spectrometer parameters were set as follows: resolution: 140,000; automatic gain control (AGC): 3 × 106; maximum IT time: 100; scan range: 72–1,200 m/z. Raw data were acquired from the Xcalibur MS software (Thermo Electron Corp, Waltham, MA) and converted to mzML format by ProteoWizard tool MSConverter (25, 26). The converted data were analyzed using MAVEN (27) and peaks were annotated with a maximum allowed error of 5 ppm. Area under the chromatographic curve was integrated based upon an in-house verified list of metabolites using exact m/z and known retention times (28). All metabolite values were normalized based on the exact mass of the tissue extracted prior to all statistical calculations.

### Data Analysis

Metabolites showing >1.5 fold differences and <0.05 *p*-value in the comparison between WB-affected and unaffected birds were considered differentially abundant. Ingenuity Pathway Analysis (IPA; Qiagen, Valencia, CA; http://www.ingenuity.com) software was used for functional annotation, canonical pathways analysis, upstream analysis, and network discovery based on human metabolome database (29). Heat maps were made by Cluster 3.0 (30) and Javatreeview 1.1 (31) using log2 transformed data. *P*-values were calculated using Student's *T*-test. Partial Least Squares Discriminant Analysis (PLS-DA) and Variable Importance in Projection (VIP) scores were constructed using the statistical package DiscriMiner in R version 3.6.1 (https://cran.r-project.org). These VIP scores provide a metric for determining how much influence a metabolite has on the group separation seen in the PLS-DA plots. A value > 1 indicates that the metabolite contributes to the group differentiation, and this was considered as a significant VIP score.

## Results

### Multivariate Analysis and Comparative Metabolomics Profile in WB-Affected and Unaffected Birds

**Figure 2:**
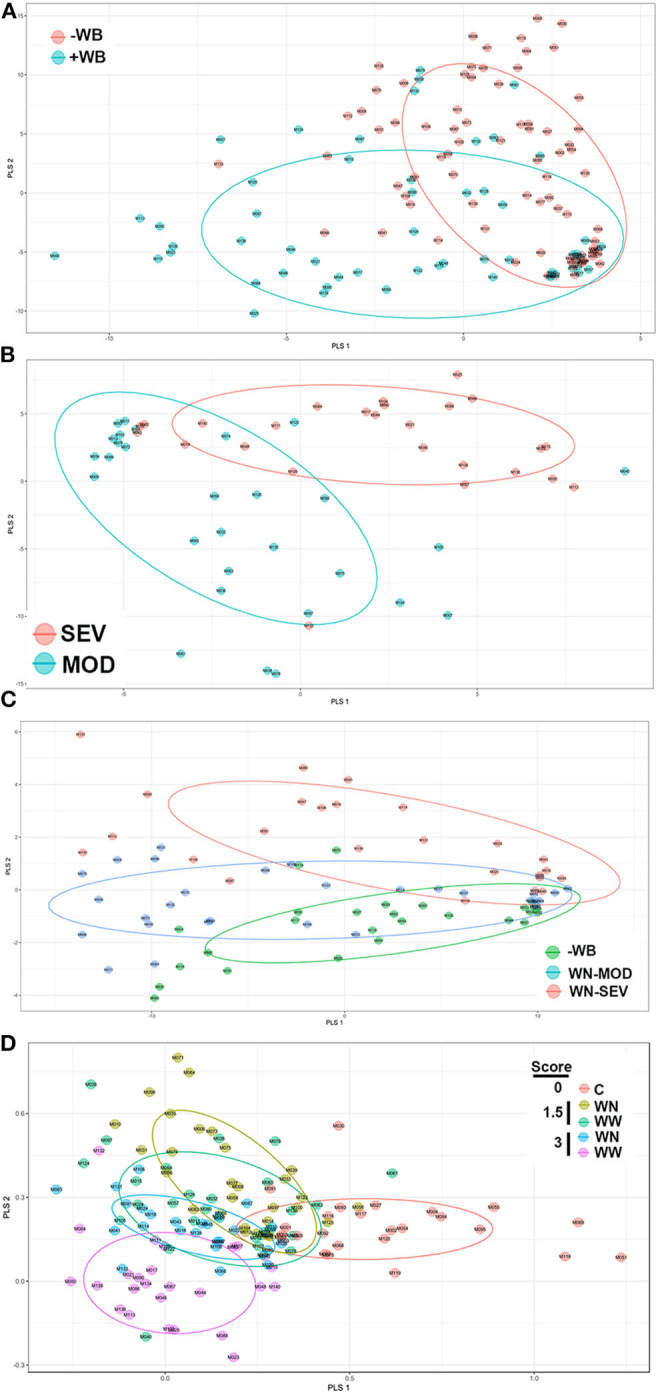
Partial Least Squares Discriminate Analysis (PLS-DA) scores plots. PLS-DA was constructed using the statistical package DiscriMiner in R version 3.6.1 and displayed different clusters when comparing metabolite profiles between WB-affected and unaffected birds **(A)**, MOD and SEV WB **(B)**, WN-MOD, and WN-SEV **(C)**, and all the groups together **(D)**.

The untargeted metabolomics profiling analyses identified 108 known metabolites and has been submitted to MetabolLights database (https://www.ebi.ac.uk/metabolights). Formal testing, pre-metabolite by two-sample *t*-tests, showed that there were 60 metabolites with differences in mean intensity at *P* < 0.05 (Table S1). Of these, 42 were at higher concentrations and 18 were at lower levels in WB-affected birds compared to their unaffected counterparts (Table S1). Most altered metabolites belonged to the nucleosides, nucleotides and analogs (35.06%), amino acids, peptides and analogs (22.95%), carbohydrates and carbohydrate conjugates (13.11%), dicarboxylic acids (4.92%), pyridines and derivatives (4.92%), and 1.64% of each of phosphate ester, furanones, organosulfonic acid, keto acids, fatty acids, hydroxyl acids, flavonoids, peptidomimetics, alcohols, amines, and lactams. When the metabolite profiles for each WB-affected and unaffected group were represented using a PLS-DA scores plot, two clusters were observed (Figure 2A). Similarly, PLS-DA analyses and score plots displayed different clusters when comparing metabolites profile from MOD and SEV WB-affected muscles (Figure 2B). Within apparent healthy muscles obtained from control S1 region, and MOD and SEV S2 regions, PLS-DA scores plots showed 3 separate clusters (Figure 2C). When all the samples were plotted together, 5 clusters were identified (Figure 2D). This indicates that a clear separation of affected- and unaffected-tissues on one hand, and WB category (MOD *vs*. SEV) on the other hand can be achieved and that unique metabolite profiles are present for the WB-affected and unaffected muscles. The PLS-DA combines features from principle component analysis and multiple regression and transforms a large number of potentially correlated variables into a smaller number of orthogonal variables (i.e., PLS1, PLS2) that discriminates between classes. In order to determine which metabolites drove the separation among the groups, each metabolite was assigned a VIP score representing its importance to the PLS-DA model with a score >1 indicating a significant contribution to the separated clusters observed. As shown in Table 1, 43 metabolites were found to be driving the separation between WB-affected and unaffected muscles, and 38 between MOD and SEV affected tissues in the PLS-DA plot. Of these, 22 common metabolites are involved in all the segregation (Table 1).

**Table 1:** Metabolites driving cluster separations based on VIP score^a^.

**Metabolites**	**C *vs*. WB**	**WN1.5 *vs*. WN3**	**WW1.5 *vs*. WW3**
Flavone	+	+	+
Glucose6phosphate	+	+	+
Tricarballylicacid	+	+	+
Guanidoaceticacid	+	+	+
Sedoheptulose1phosphate	+	+	+
UTP	+	+	+
3−Phosphoglycerate	+	+	+
GDP	+	−	+
Histamine	+	+	+
NAD+	+	−	+
Deoxycytidine	+	+	+
Trehalose/Sucrose	+	+	−
2, 3−Biphosphoglycerate	+	+	−
Homocamosine	+	−	+
Vanillin	+	−	−
Phosphorylethanolamine	+	+	+
AMP/dGMP	+	+	−
Salicylate	+	+	+
Cytidine	+	+	−
UDP−N−acetylglucosamine	+	+	+
Myo−inositol	+	+	+
Methylsuccinicacid	+	+	−
Cysteine	+	−	+
Glutaricacid	+	+	−
CDP−ethanolamine	+	+	+
Cystathionine	+	+	+
2−Oxoisovalerate	+	+	+
4−Pyridoxoate	+	+	+
2−Isopropylmatate	+	+	+
S−Adenosyl−L−homocysteine	+	+	−
Dephospho−CoA	+	+	+
2−Dehydro−D−gluconate	+	−	−
Glucosaminephosphate	+	−	+
Deoxyuridine	+	−	+
Alpha−Ketoglutarate	+	−	+
Citrulline	+	+	+
Hydroxypherrylpyruvate	+	+	+
Homocysteine	+	+	+
Shikimate	+	−	+
Pyroglutamicacid	+	−	−
Dimethylglycine	+	−	+
NADH	+	−	+
Ophthalmate	+	−	−
Taurine	−	+	+
ADP	−	+	+
AICAR	−	+	−
CDP	−	+	+
Proline	−	+	−
NADH	−	+	−
Glycerate	−	+	−
Dopamine	−	+	−
1−Methyladenosine	−	+	+
Deoxyribosephosphate	−	+	+
N−Carbamoyl−L−aspartate	−	−	+
Flavone	+	+	+
Glucose 6 phosphate	+	+	+
Tricarballylic acid	+	+	+
Guanidoacetic acid	+	+	+
Sedoheptulose 1 phosphate	+	+	+
UTP	+	+	+
3-Phosphoglycerate	+	+	+
GDP	+	−	+
Histamine	+	+	+
NAD+	+	−	+
Deoxycytidine	+	+	+
Trehalose/Sucrose	+	+	−
2,3-Biphosphoglycerate	+	+	−
Homocamosine	+	−	+
Vanillin	+	−	−
Phosphorylethanolamine	+	+	+
AMP/dGMP	+	+	−
Salicylate	+	+	+
Cytidine	+	+	−
UDP-N-acetylglucosamine	+	+	+
Myo-inositol	+	+	+
Methyl succinic acid	+	+	−
Cysteine	+	−	+
Glutaric acid	+	+	−
CDP-ethanolamine	+	+	+
Cystathionine	+	+	+
2-Oxoisovalerate	+	+	+
4-Pyridoxoate	+	+	+
2-Isopropylmatate	+	+	+
S-Adenosyl-L-homocysteine	+	+	−
Dephospho-CoA	+	+	+
2-Dehydro-D-gluconate	+	−	−
Glucosamine phosphate	+	−	+
Deoxyuridine	+	−	+
Alpha-Ketoglutarate	+	−	+
Citrulline	+	+	+
Hydroxypherrylpyruvate	+	+	+
Homocysteine	+	+	+
Shikimate	+	−	+
Pyroglutamic acid	+	−	−
Dimethylglycine	+	−	+
NADH	+	−	+
Ophthalmate	+	−	−
Taurine	−	+	+
ADP	−	+	+
AICAR	−	+	−
CDP	−	+	+
Proline	−	+	−
NADH	−	+	−
Glycerate	−	+	−
Dopamine	−	+	−
1-Methyladenosine	−	+	+
Deoxyribose phosphate	−	+	+ +
N-Carbamoyl-L-aspartate	−	−	+

a *VIP, Variable Importance in Projections scores were obtained from PLSDA using the DiscriMiner software package. +, indicates a VIP score >1 and − indicates a VIP score <1*.

### Metabolic and Canonical Pathways Analysis

**Figure 3:**
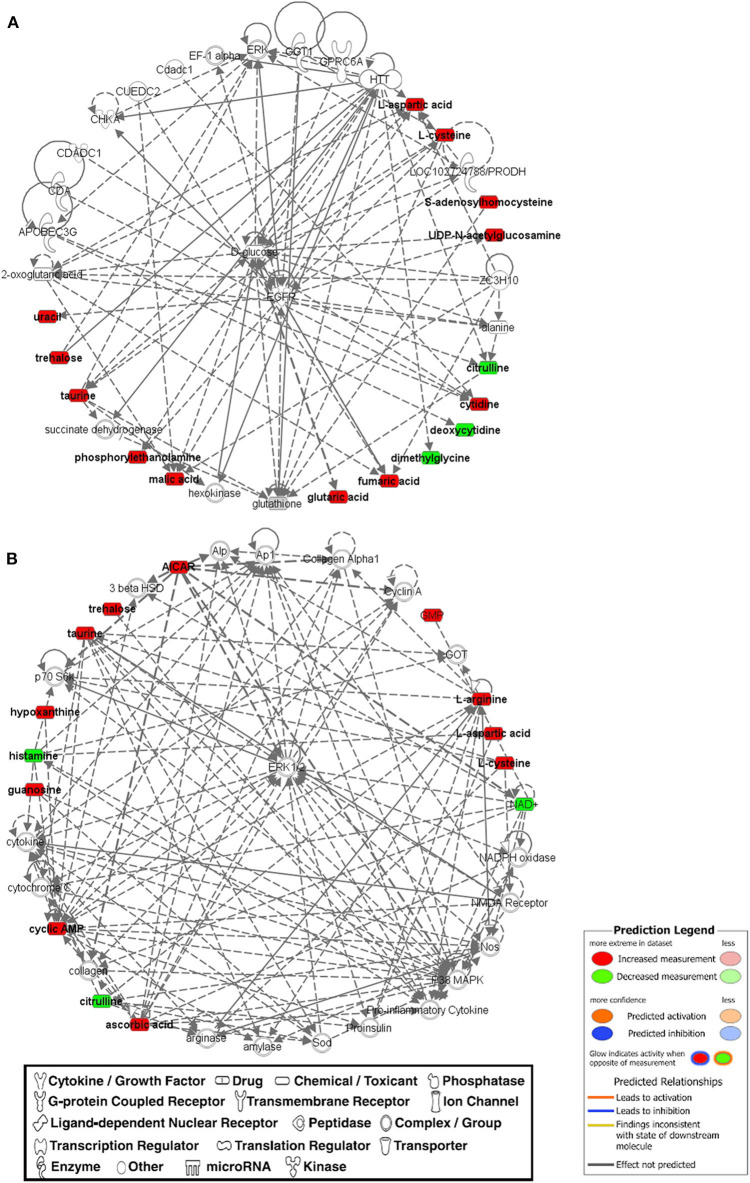
The top two networks **(A,B)** built with IPA program from metabolomics data that was determined on WB-affected muscles. Metabolites in red were upregulated and metabolites in green were down-regulated in WB-affected tissues. Networks constructed from the IPA knowledgebase by connecting the altered molecules are not limited by canonical pathway boundaries. EGFR, epidermal growth factor receptor; ERK, extracellular signal-regulated kinase.

For more stringent pathway analyses and to gain biologically related molecule networks, the above identified metabolites (108) were mapped into the IPA knowledge-base and analyzed to outline the most enriched biological functions. Using a cut-off of FDR adjusted *p*-value < 0.05 and a fold-change between −1.5 and 1.5, IPA analysis identified 50 differentially abundant (DA) metabolites between WB-affected and unaffected birds (Table 2). Among the 50 DA metabolites, 37 were significantly higher and 13 were significantly lower in the breast muscle of WB-affected birds compared to their unaffected counterparts (Table 2). The top 10 canonical pathways generated with DA-metabolites were purine nucleotide degradation and *de novo* biosynthesis, sirtuin signaling pathway, citrulline-nitric oxide cycle, salvage pathways of pyrimidine DNA, IL-1 signaling, iNOS, Angiogenesis, PI3K/AKT signaling, and oxidative phosphorylation (Table 3). The top altered bio-functions in term of molecular and cellular functions in breast muscle comparison between WB-affected and unaffected birds included cellular development, cellular growth and proliferation, cellular death and survival, small molecular biochemistry, inflammatory response, free radical scavenging, cell signaling and cell-to-cell interaction, cell cycles, and lipid, carbohydrate, amino acid, and nucleic acid metabolisms (Table 4). The top disease and disorder functions identified by IPA analysis were organismal injury and abnormalities, cancer, skeletal and muscular disorders, connective tissue disorders, and inflammatory diseases (Table 4). According to the canonical pathway analysis, 13 prediction networks were identified. The first top predicted network used 15 metabolites and focuses on amino acid metabolism, carbohydrate metabolism, molecular transport, and small molecules biochemistry (Figure 3). Metabolites like S-adenosylhomocysteine, UDP-N-acetylglucosamine, L-cysteine, trehalose, and deoxycytidine that characterized by 11, 5.34, 3.84, 2.83, and −3.32-fold changes in WB-affected compared to unaffected tissues, had hubs at the signaling molecule epidermal growth factor receptor (EGFR) (Figure 3A). EGFR is reported to be involved in cancer, apoptosis, and skeletal and muscular disorders (32–34). Network 2 used 14 molecules and centers on energy production, amino acid metabolism, and small molecule biochemistry and has a nexus to the signaling molecule extracellular-signal-related kinase 1/2 (Figure 3B) which has been shown to play pivotal roles in cancer, cell death, and muscle disorders (35, 36).

**Table 2:** Differentially abundant metabolites in woody breast-affected and unaffected birds.

**HMDB ID**	**Metabolite name**	**Fold change**	***P*-value**
HMDB0001517	AICAR	3.255	0.000153
HMDB0000044	Ascorbic acid	3.101	0.000789
HMDB0001564	CDP ethanolamine	3.239	0.0000712
HMDB0000058	Cyclic AMP	1.898	0.0442
HMDB0000089	Cytidine	2.782	0.00021
HMDB0011732	D-2-ketogluconic acid	3.49	0.000973
HMDB0000625	D-gluconic acid	3.012	0.00353
HMDB0061388	Dimethyl-alpha-ketoglutarate	2.749	0.0218
HMDB0060475	DL-glutamic acid	1.694	0.0245
HMDB0001227	dTMP	3.456	0.000453
HMDB0001409	dUMP	3.443	0.000214
HMDB0000134	Fumaric acid	2.086	0.0389
HMDB0000661	Glutaric acid	2.003	0.0426
HMDB0001397	GMP	2.149	0.00243
HMDB0000133	Guanosine	2.937	0.0000876
HMDB0000157	Hypoxanthine	2.216	0.00439
HMDB0000517	L-arginine	1.69	0.0159
HMDB0000191	L-aspartic acid	1.875	0.0189
HMDB0000099	L-cystathionine	4.576	0.000028
HMDB0000574	L-cysteine	3.84	0.000237
HMDB0000744	Malic acid	1.969	0.0341
HMDB0001844	Methylsuccinic acid	1.98	0.0432
HMDB0001138	N-acetyl-L-glutamate	3.838	0.000725
HMDB0006029	N-acetyl-l-glutamine	2.666	0.012
HMDB0000828	N-carbamoyl-L-aspartic acid	3.059	0.0363
HMDB0005765	Ophthalmic acid	2.193	0.0062
HMDB0000224	Phosphorylethanolamine	6.265	0.0000338
HMDB0000267	Pyroglutamic acid	2.924	0.00764
HMDB0000939	S-adenosylhomocysteine	2.439	0.000472
HMDB0060509	Sedoheptulose 1-phosphate	11	0.0000145
HMDB0000251	Taurine	3.077	0.0000885
HMDB0000975	Trehalose	2.831	0.0116
HMDB0000290	UDP-N-acetylglucosamine	5.345	0.0000258
HMDB0000300	Uracil	2.117	0.0148
HMDB0000296	Uridine	2.143	0.00181
HMDB0000299	Xanthosine	2.231	0.00221
HMDB0001554	Xanthosine monophosphate	2.443	0.00699
HMDB0031193	Tricarballylic acid	−5.057	0.0243
HMDB0000904	Citrulline	−2.255	0.00496
HMDB0060465	AMP	−1.983	0.00846
HMDB0000014	Deoxycytidine	−3.325	0.0022
HMDB0000092	Dimethylglycine	−1.932	0.0161
HMDB0003075	Flavone	−18.384	0.0106
HMDB0001201	GDP	−2.283	0.0248
HMDB0001254	Glucosamine phosphate	−2.437	0.0171
HMDB0001401	Glucose-6-phosphate	−9.383	0.000114
HMDB0000128	Guanidoacetic acid	−5.852	0.0229
HMDB0000870	Histamine	−2.165	0.0104
HMDB0000745	Homocarnosine	−2.414	0.00113
HMDB0000902	NAD+	−2.809	0.00122

**Table 3:** Top canonical pathways enriched by observed metabolite alteration in WB myopathy.

**Canonical pathways**	**Molecules**	**-log (*p*-value)**	**Ratio**
Purine nucleotides degradation II	GMP, guanosine, hypoxanthine, NAD, xanthosine, xanthosine monophosphate	6.00	0.353
Purine nucleotides *de novo* biosynthesis II	AICAR, fumaric acid, GDP, GMP, L-aspartic acid, NAD, xanthosine monophosphate	5.58	0.233
Sirtuin signaling pathway	Citrulline, fumaric acid, glucose-6-phosphate, L-aspartic acid, malic acid, NAD	4.16	0.182
Citrulline-nitric oxide cycle	Citrulline, fumaric acid, L-arginine, L-aspartic acid	4.16	0.364
Salvage pathways of pyrimidine deoxyribonucleotides	Deoxycytidine, dTMP, dUMP, uracil	3.99	0.333
IL-1 signaling	Cyclic AMP, GDP	2.82	0.667
iNOS signaling	Citrulline, L-arginine	2.82	0.667
Inhibition of angiogenesis	Citrulline, L-arginine	2.31	0.400
PI3K/AKT signaling	Citrulline, L-arginine	2.31	0.400
Oxidative phosphorylation	Fumaric acid, NAD	1.69	0.200

**Table 4:** Top diseases, molecular, cellular, and physiological functions for DE-metabolites in WB-affected compared to unaffected birds.

**Diseases and functions**	***P*-value**	# Molecules^*^
Organismal injury and abnormalities	2.55^E−06^-4.54^E−02^	26
Cancer	2.55^E−06^-3.93^E−02^	20
Skeletal and muscular disorders	6.09^E−03^-3.41^E−02^	10
Connective tissue disorders	6.09^E−03^-3.41^E−02^	6
Inflammatory diseases	6.09^E−03^-4.54^E−02^	6
**Molecular and Cellular Functions**	***P*****-value**	# Molecules^*^
Small molecule biochemistry	1.46^E−04^-4.54^E−02^	23
Cellular growth and proliferation	3.38^E−05^-4.47^E−02^	21
Cellular death and survival	3.68^E−05^-4.54^E−02^	19
Molecular transport	1.46^E−04^-4.54^E−02^	19
Cellular development	3.38^E−05^-4.47^E−02^	17
Lipid metabolism	1.46^E−04^-4.54^E−02^	17
Inflammatory response	1.52^E−03^-4.54^E−02^	17
Cellular function and maintenance	3.68^E−04^-4.22^E−02^	15
Free radical scavenging	1.46^E−04^-3.03^E−02^	14
Carbohydrate metabolism	1.20^E−03^-4.54^E−02^	14
Nucleic acid metabolism	3.37^E−03^-4.54^E−02^	13
Cellular assembly and organization	3.68^E−04^-3.79^E−02^	12
Connective tissue development and function	3.90^E−05^-4.54^E−02^	12
Cell morphology	3.68^E−04^-4.47^E−02^	11
Amino acid metabolism	6.67^E−04^-4.47^E−02^	11
Cell signaling	6.80^E−03^-3.79^E−02^	10
Cell-to-cell signaling and interaction	3.68^E−04^-4.54^E−02^	10
Protein synthesis	4.82^E−03^-3.41^E−02^	9
Vitamin and mineral metabolism	6.80^E−03^-3.41^E−02^	6
Cell cycle	2.99^E−03^-4.36^E−02^	7
Immune cell trafficking	3.37^E−03^-4.54^E−02^	7
Skeletal and muscular system development and function	7.27^E−06^-4.47^E−02^	7
Energy production	2.99^E−03^-4.36^E−02^	6

* *Number of molecules with each network. The molecule networks were generated from association with IPA database and our input data. The P-value was calculated using Fisher's exact Test by IPA*.

### Effect of QB on WB Incidence and Muscle Metabolome Profile

**Figure 4:**
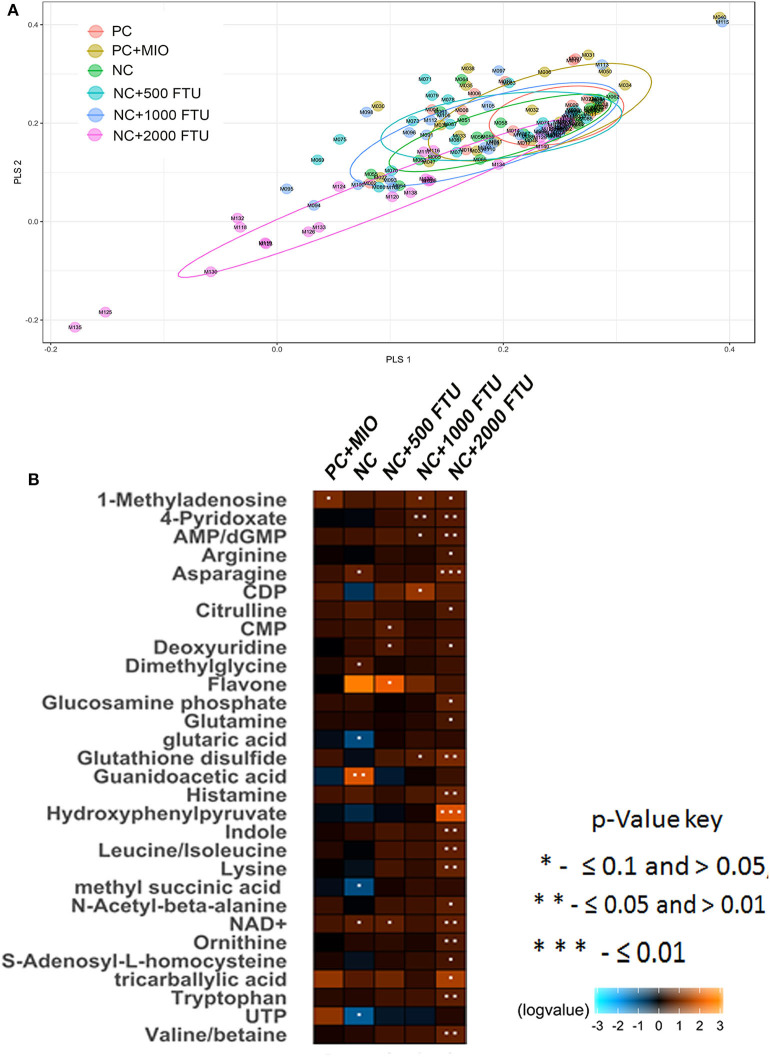
Partial Least Squares Discriminate Analysis (PLS-DA) scores plots and heat maps of relative levels of most significant metabolites modulated by high dose quantum blue-enriched diets. **(A)** PLS-DA was constructed using the statistical package DiscriMiner in R version 3.6.1 and displayed different clusters when comparing metabolite profiles between all diets, **(B)** heat maps was build using log2 ratio and VIP score and comparing each diet to the PC group.

QB supplementation at high doses (1,000 and 2,000 FTU) reduced the severity of WB by ~5% compared to the positive control [Table 5, (22)]. Multivariate data analysis and PLS-DA score plots displayed 6 different clusters when comparing all diet supplementations (Figure 4A). Samples from birds fed with high dose of QB (NC+2,000 FTU) form a distinct and segregated cluster from the rest of the groups (Figure 4A). Further analysis using VIP scores revealed that 30 metabolites drove the separation among the groups. As illustrated in Figure 4B and Figure S1, heat maps showed the relative levels of metabolites in each group compared to the PC control. Breast tissues from QB (2,000 FTU)-fed birds exhibited 22 metabolites with significantly different levels compared to the PC group (Figure 4B). These metabolites belong to amino acids (47.80%), nucleosides (26.13%), carboxylic acids (8.65), carbohydrates (4.35%), phenylpyruvic acids (4.37%), amines (4.35), and indoles (4.35%). Of these 22 metabolites, only two (S-adenosyl-L-homocysteine and arginine) were found to be significantly higher and seven (tricarballylic acid, NAD^+^, glucosamine phosphate, citrulline, histamine, AMP/dGMP, and 4-pyridoxate) were found to be significantly lower in WB-affected compared to unaffected birds.

**Table 5:** Effects of quantum blue supplementation on WB incidence (%) in broilers.

**Parameters^**a**^**	**Diets**					
	**PC**	**PC+MIO**	**NC**	**NC+500 FTU**	**NC+1,000 FTU**	**NC+2,000 FTU**
**WB category**^**b**^						
NORM	10.34	8.19	24.24	5.08	5.68	10.54
MOD	68.96	68.85	69.69	71.18	78.42	73.68
SEV	20.68	22.95	6.07	23.74	15.90	15.78
Total incidence	89.64	91.8	75.76	94.92	94.31	89.46

a *The scoring and incidence of WB were determined using 8 replicate pens/treatment and 8 birds/pen*.

b *MOD, moderate; NORM, normal; SEV, severe*.

## Discussion

WB is a stumbling block and significant economic burden to the poultry industry worldwide and for which there is no effective preventive strategy due to its unknown etiology. Several elegant high-throughput omics studies predicted the involvement of various pathways (18, 21, 37) and several seminal managerial and nutritional strategies including our own were conducted to reduce the severity of WB myopathy (22, 38, 39). Because metabolites play a key role in the maintenance and growth of organisms and are considerably useful to illustrate the underlying molecular disease-causing mechanisms (40, 41), we used here a metabolomics approach to identify key metabolite markers and outline the most enriched biological functions in WB myopathy.

**Figure 5:**
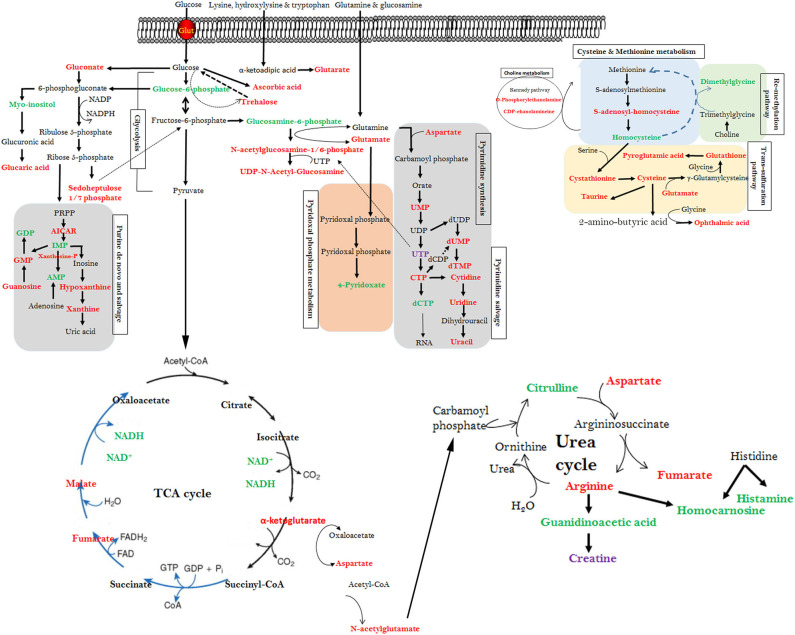
Interconnected altered metabolic pathways in WB-affected muscles. Altered metabolites from the top canonical pathway analysis along with their cross-independence in KEGG pathways. Red text metabolites represent elevated mean intensity in WB-affected, whereas green text represent reduced intensity. The figure is representative and not for scale.

Our high-throughput metabolomics analysis and PLS-DA scores plots demonstrated that distinct metabolic profiles can be detected for WB-affected and unaffected tissues. The altered metabolites encompassed all major molecular categories and critical metabolic pathways from amino sugar and nucleotide sugar metabolism, methionine and choline metabolism, and purine/pyrimidine salvage to Krebs and urea cycles. Arranging these DA metabolites using both IPA and KEGG metabolic pathways into a single map revealed a high level of connectivity and inter-dependence (summarized in Figure 5).

Of particular interest and in agreement with previous study (8), the 10-fold decrease in glucose-6-phosphate indicates a potential dysregulation of both the glycolysis and glycogenesis in WB-affected muscle. It has been shown that a defect in glucose-6-phosphate concentration was a major contributor to reduce whole-body insulin-mediated glucose disposal rates and to reduce insulin action on glycogen synthase (42). Although it was not measured in the present study, dysregulation of glucose-6-phosphate levels indicated an alteration of glycogen content in muscle with WB (8, 43). This is supported here by the increased levels of AICAR, which has been reported to alter glycogen content via alteration of glycogen synthase and glycogen phosphorylase activities (44). AICAR is an analog of adenosine monophosphate and is capable of stimulating the master energy sensor, AMP-dependent protein kinase (AMPK), which is higher in WB-affected compared to unaffected muscles (data not shown) indicating an intracellular ATP depletion status. AICAR plays a crucial role in the recruitment of ATP-sensitive K channels to the sarcolemma and the regulation of calcium content (45), and calcium overloaded was found to be a hallmark of muscle dystrophies (46). AICAR has also been shown to alter mTOR signaling pathway in skeletal muscle (47) and our previous study demonstrated that WB myopathy is associated with mTOR dysmetabolism-induced hypoxia (22).

The dysregulation of glucose metabolism (reduction of glucose-6-phosphate) was accompanied by an accumulation of trehalose, ascorbate, and gluconate in WB-affected tissues. The presence of trehalose is surprising as it has not been found in higher vertebrate to date even though the enzyme trehalase required for trehalose hydrolysis has been reported (48). Trehalose is synthesized as a stress-responsive factor and has been implicated in cyto-protection against hypoxia, neurodegenerative disease, and oculopharyngeal muscular dystrophy (49–51). The glucose-derived molecule ascorbate functions as an enzyme cofactor and antioxidant, and ~40% of the body's ascorbate is stored in skeletal muscle (52). However, its significant increase in our experimental conditions suggested that this might due to excess of oxidative stress in WB-affected muscles (8). As high doses of antioxidants interfere with muscle signaling pathways and can be detrimental (53), it is possible that high concentration of ascorbate along with low concentration of flavone (−18 fold changes) might exacerbate the WB myopathy. The alteration of gluconate metabolism (increase of gluconate and 2-keto-L-gluconate levels) in WB suggested an activation of amino acid catabolism in order to provide carbon skeleton for anaplerosis which is evident here by increased concentrations of glutamate (converted to α-ketoglutarate) and aspartate (converted to oxaloacetate), main components in TCA cycle. The increased levels of α-ketoglutarate, fumarate, and malate in this study suggested a dysregulated cataplerosis pathway resulting in accumulation of these metabolites in the mitochondrial matrix. Interestingly, one of the 2-keto-L-gluconate binding target was the β-subunit of the mitochondrial ATP synthase resulting in reduced mitochondrial oxygen consumption and oxidative phosphorylation (54), which has been predicted by IPA analysis in this study. Furthermore, 2-keto-L-gluconate has been shown to regulate acid-base balance (55), which could be the reason for higher ultimate pH seen in WB-affected muscles (37). The accumulation of the above mentioned metabolites in association with mitochondrial dysfunction can lead to high levels of glucaric (4-fold changes) and glutaric acids (2-fold changes). Glucaric and glucoronic acids can act as acidogen and metabotoxin and their accumulation has been reported to cause damage to several organs via up-regulation of the production and release of endogenous inflammatory signaling molecules (56). Glutaric acid administration has been shown to alter mitochondrial complexes I-III and II-III and impair energy metabolism in rodent skeletal muscle (57).

The pentose phosphate pathway (PPP), paralleling an approximate 11-fold increase in sedoheptulose 1/7 phosphate, was altered in WB-affected tissues. Previous studies showed that hydrogen peroxide treatments elevated sedoheptulose 1/7 phosphate levels in hepatoma HepG2 cells (58). Because the PPP activation was reported to be involved in cyto-protection against oxidative damage (59), our data suggested that oxidative stress might increase the flux of glucose into the PPP pathway. Consistent with an altered redox homeostasis, the levels of antioxidant-associated metabolites was affected. For instance, the major low molecular weight antioxidant glutathione as well as its trans-sulfuration sources were increased in WB-affected tissues suggesting high metabolic stress (peroxides and free radicals) and intracellular detoxification of xenobiotics and cell damaging compounds (60). Our data suggested also that chicken muscle cells might serve as a continuous extrahepatic source of glutathione during local trauma where the release of stress hormone may greatly enhance the release of the antioxidant (61). Furthermore, the aminoethane sulfonic acid, taurine, was higher in WB-affected compared to unaffected muscles. In addition to its antioxidant activity, taurine was found to be involved in mitochondrial t-RNA and mitochondrial protein synthesis (62). Taurine also plays a crucial role in calcium homeostasis and muscle contractile function (63, 64), both of which were defective in WB myopathy.

The alteration of methionine and choline metabolism was demonstrated by higher levels of s-adenosyl-homocysteine (SAH), phosphorylethanolamine and CDP-ethanolamine in WB-affected tissues. S-adenosyl-L-homocysteine is the major donor of methyl groups in the synthesis of phospholipids, nucleotides, epinephrine, carnitine, and creatine. It has been reported that, when present at high levels, SAH can act as an immunotoxin (disrupts, limits, or destroys the function of immune cells) and it has been linked to metabolic alteration, cardiovascular disease, and end-stage renal disease (65, 66). Phosphorylethanolamine was found to be linked to oxidative stress in cultured rat lenses (67). The diacylglycerol-consuming CDP-ethanolamine pathway is a major route for phosphatidylethanolamine synthesis, which constitutes the second major abundant phospholipids (68). In addition to controlling muscle diacylglycerol levels, emerging evidence indicated that CDP-ethanolamine pathway plays a significant role in regulating muscle function and mitochondrial biology (69).

A noteworthy additional point is the alteration of the nucleotide (purine and pyrimidine) *de novo* synthesis, catabolism, and salvage. These processes are energy-expensive, but they are crucial for every living cell, and their alteration may lead to significant functional consequences. For example, guanosine, which plays a key role in muscle contraction and signaling pathways (70), has been reported to be toxic at high concentration (71). High levels of serum uracil concentration has been used as a predictor of severe fluoropyrimidine-associated toxicity (72). Similarly high concentrations of xanthine and hypoxanthine have been found to be toxic (73, 74). Because hypoxanthine is very low under physiological conditions, our data suggested that hypoxanthine was build up with the progression of WB myopathy with concomitant production of reactive oxygen species (75, 76). Its high levels have been shown to be associated with muscle damage (77). The alteration of the aforementioned metabolic pathways in WB myopathy lead probably to excessive consumption of ATP and NAD^+^ which is supported by the activation of AMPK (data not shown) and a decreased levels of NAD^+^ (our metabolomics data), respectively. It has been shown that oxidative stress induced a deficit in NAD^+^ which in turn promoted generation of methylglyoxal that glycated nucleic acid and protein and conduced to advanced glycation end product (AGE) formation (78, 79).

While some of our altered metabolites have been reported by Abasht and co-workers (for fumarate, malate, sedoheptulose 1/7 phosphate, glutathione, taurine, and nucleotides) (8), by Wang and colleagues (for taurine, hypoxanthine, and NAD^+^) (20), and by Soglia et al. (80) (for taurine, uracil, and hypoxanthine), several metabolites such as AICAR, trehalose, flavones, etc. constitute new molecular signatures, and they have not been reported previously. The common altered metabolites observed in all three studies might constitute conserved metabolic pathways in WB myopathy across all broiler strains. The distinct metabolites observed in our study might be strain- or age-specific as we used 56d-old Cobb500 compared to 38d-old Ross708 by Wang et al. (20), and 47-48d-old undisclosed purebred lines and commercial broiler by Abasht et al. (8). Furthermore, these studies used different metabolomics-based methods [for example NMR in Wang's (20) vs. UPLC–HRMS in this study]. The effects of intrinsic sensitivity of detection and the capacity of separation of complex metabolite mixture by these different methods are not ruled out.

Ameliorating WB incidence by reducing its severity is of uppermost interest to the poultry industry because it would help in improving bird welfare, meat quality, and overall poultry production sustainability. As we previously showed that QB supplementation reduced the severity of WB myopathy (22), we sought to determine here its effect on breast muscle metabolomics profiling. Our data suggested that QB might improve WB via modulation of s-adenosyl-homocystein, arginine, tricarballylic acid, NAD^+^, glucosamine phosphate, citrulline, histamine, AMP, and pyridoxate. Although the exact underlying mechanisms are not known at this time, it is possible that QB modulates the above mentioned metabolites via improved solubility and digestibility of dietary nutrients and thereby enhanced release and bioavailability of calcium, phosphorus, minerals, and metal co-factors for endogenous enzymes and inositol liberation (81). For instance, it has been shown that QB superdosing improved liberation of zinc (82) and zinc has been reported to interact with ATP/ADP/AMP-hydrolyzing enzymes and have an antioxidant role via protection of sulfhydryl group against oxidation and inhibition of the ROS production by transition metals (83). Similarly, Tang and co-workers showed that phosphorus had antioxidant properties via enhancing superoxide dismutase and catalase activities in yellow cat fish (84). In chickens, it has been demonstrated that QB superdosing increased the levels of coenzyme Q(10), retinol, and alpha-tocopherol in liver (85). As coenzyme Q(10), retinol, and alpha-tocopherol all have been shown to improve mitochondrial respiration and function (86–88), our data suggested that QB might reduce the severity of WB via an improvement in the muscle oxidative status and metabolic profile.

In summary, we determined many metabolic biomarkers and disordered pathways, which could be regarded as new routes for discovering potential mechanisms of WB myopathy. Further in-depth investigations are warranted to define the mechanisms by which QB phytase modulates the metabolomics profile-ameliorating WB incidence.

## Data Availability Statement

The datasets presented in this study can be found in online repositories. The names of the repository/repositories and accession number(s) can be found in the article/Supplementary Material.

## Ethics Statement

The animal study was reviewed and approved by University of Arkansas.

## Author Contributions

SD conceived and designed the study. EG, RC, AD, and SD conducted the experiments and analyzed the data. SH, HC, and SC performed the UPLC-HRMS and generated the metabolomics data. MB provided the QB. SD wrote the paper with a critical review by MB, BK, and MK. All authors contributed to the article and approved the submitted version.

## Conflict of Interest

MB is employed by company AB Vista. The remaining authors declare that the research was conducted in the absence of any commercial or financial relationships that could be construed as a potential conflict of interest.
