# Supplementary material for: Muscle Metabolome Profiles in Woody Breast-(un)Affected Broilers: Effects of Quantum Blue Phytase-Enriched Diet
Source: Front Vet Sci. 2020 Aug 4;7:458. doi: 10.3389/fvets.2020.00458 (PMC7417653; doi:10.3389/fvets.2020.00458)
Supplement: Supplementary file 1 [file Table_1.DOCX]

**Table S1.** Untargeted metabolomics profiling analyses of breast muscle from WB-affected and unaffected birds^1, 2^.

| **Metabolites** | **HMDB ID** | **Fold changes** | ***P*-Value** | **Class** |
| --- | --- | --- | --- | --- |
| Sedoheptulose 1-phosphate  O-Phosphorylethanolamine  UDP-N-acetylglucosamine  Cystathionine  Glucaric acid  N-Acetylglutamate  Cysteine  N-Acetylglucosamine 1/6-phosphate  2-Dehydro-D-gluconate  dTMP  dUMP  AICAR  CDP-ethanolamine  Ascorbic acid  Taurine  D-Gluconate  N-Carbamoyl-L-aspartate  Guanosine  Pyroglutamic acid  Trehalose/Sucrose  Cytidine  alpha-Ketoglutarate  N-Acetylglutamine  Xanthosine 5-phosphate  S-Adenosyl-L-homocysteine  Xanthosine  Hypoxanthine  GMP  Uridine  Ophthalmate  Uracil  Fumarate  Glutaric acid  methyl succinic acid  Malate  cAMP  Aspartate  Nicotinate  Glutamate  Arginine  UMP  Nicotinamide  Glutathione  Flavone  Glucose 6 phosphate  Guanidoacetic acid  tricarballylic acid  deoxycytidine  NAD+  Glucosamine phosphate  homocarnosine  Homocysteine  GDP  Citrulline  myo-Inositol  Histamine  AMP  Dimethylglycine  NADH  IMP  4-Pyridoxate  Indole  Inosine  Leucine/Isoleucine  Lysine  N-Acetyl-beta-alanine  N-Acetylornithine  Ornithine  Pantothenate  Proline  Salicylate  Serine  Shikimate  Succinate/Methylmalonate  Thymine  Tryptophan  Tyrosine  UTP  Valine/betaine  Vanillin  1-Methyladenosine  2,3-Bisphosphoglycerate  2-hydroxyglutaric acid  2-Isopropylmalate  2-Oxoisovalerate  3-Hydroxyisovalerate  3-Phosphoglycerate  Acetyllysine  ADP  Asparagine  CDP  CMP  Creatine  dAMP  Deoxyadenosine  Deoxyribose phosphate  Deoxyuridine  Dephospho-CoA  Dopamine  Glutamine  Glutathione disulfide  Glycerate  Homoserine/Threonine  Homovanillic acid (HVA)  hydroxybutyrate  Hydroxyisocaproic acid  Hydroxyphenylpyruvate  Hydroxyproline | HMDB0060509  HMDB0000224  HMDB0000290  HMDB0000099  HMDB0000663  HMDB0001138  HMDB0000574  HMDB0002817  HMDB0011732  HMDB0001227  HMDB0001409  HMDB0001517  HMDB0001564  HMDB0000044  HMDB0000251  HMDB0000625  HMDB0000828  HMDB0000133  HMDB0000267  HMDB0000975  HMDB0000089  HMDB0061388  HMDB0006029  HMDB0001554  HMDB0000939  HMDB0000299  HMDB0000157  HMDB0001397  HMDB0000296  HMDB0005765  HMDB0000300  HMDB0000134  HMDB0000661  HMDB0001844  HMDB0000744  HMDB0000058  HMDB0000191  HMDB0001488  HMDB0060475  HMDB0000517  HMDB0000288  HMDB0001406  HMDB0000125  HMDB0003075  HMDB0001401  HMDB0000128  HMDB0031193  HMDB0000014  HMDB0000902  HMDB0001254  HMDB0000745  HMDB0000742  HMDB0001201  HMDB0000904  HMDB0000211  HMDB0000870  HMDB0060465  HMDB0000092  HMDB0001487  HMDB0015536  HMDB0000017  HMDB0000738  HMDB0000195  HMDB0028932  HMDB0003405  HMDB0061880  HMDB0003357  HMDB0000214  HMDB0000210  HMDB0000162  HMDB0000500  HMDB0000187  HMDB0003070  HMDB0000202  HMDB0000262  HMDB0000929  HMDB0000158  HMDB0000285  HMDB0240571  HMDB0012308  HMDB0003331  HMDB0001294  HMDB0000694  HMDB0000402  HMDB0000407  HMDB0000754  HMDB0000807  HMDB0000206  HMDB0000061  HMDB0000168  HMDB0001546  HMDB0000095  HMDB0000064  HMDB0000905  HMDB0000101  HMDB0001031  HMDB0000012  HMDB0001373  HMDB0000073  HMDB0003423  HMDB0003337  HMDB0000139  HMDB0000719  HMDB0000118  HMDB0000710  HMDB0000746  HMDB0011663  HMDB0000725 | 10.99959495  6.264567181  5.34535845  4.576093579  4.216097433  3.838137024  3.840433199  3.51781643  3.490426868  3.456485947  3.442526553  3.254871287  3.238813768  3.100819651  3.076741977  3.012149083  3.058657605  2.936993679  2.923714001  2.831263568  2.782180161  2.748963404  2.665652513  2.442951999  2.438933  2.231154994  2.215717675  2.148961413  2.142725209  2.193118525  2.117430648  2.086101451  2.002867742  1.979941176  1.968758138  1.898214606  1.874577251  1.734351928  1.693953035  1.689548641  1.637224903  1.617350783  1.47002004  -18.38358811  -9.383446733  -5.851676973  -5.056663578  -3.325386291  -2.809353853  -2.437073653  -2.41440859  -2.386072061  -2.282731506  -2.254805837  -2.249753455  -2.164545907  -1.983459033  -1.93170089  -1.720985837  -1.46949852  -1.458266258  0.953881824  1.253378038  1.24281274  1.067968453  1.067196725  1.43174889  1.237542341  1.156780608  0.714947437  0.994320629  0.903930892  0.992013959  1.146622944  0.919052919  1.092084869  1.200242001  0.798299059  0.993426611  1.219971436  1.320627947  0.086629038  1.521471095  1.047879789  1.270919057  0.738694074  2.240401986  1.252879788  1.160904373  0.930340282  1.218555309  1.235378782  0.667666411  1.303046316  1.1629418  0.481435611  1.102813164  0.749095614  1.341036546  0.997112661  1.224753468  1.109011593  0.689594944  1.127404537  1.349711808  0.782545869  1.816630067  1.177863271 | 1.45396E-05  3.37642E-05  2.58279E-05  2.80303E-05  0.001242503  0.000725321  0.000236825  0.000116931  0.000972692  0.000452774  0.000213585  0.000153059  7.11707E-05  0.000788877  8.85373E-05  0.003527546  0.03630691  8.76354E-05  0.007636308  0.011597298  0.000210359  0.02181193  0.01202883  0.006987661  0.00047237  0.002212703  0.004393255  0.002432395  0.001813056  0.006195167  0.014824316  0.038899732  0.042608382  0.043173556  0.034131751  0.044193289  0.018913953  0.082468839  0.024479834  0.01591444  0.057941095  0.087782058  0.069250103  0.010595989  0.000114318  0.022903401  0.024315356  0.002197796  0.001215598  0.017082114  0.00112846  0.087585313  0.024784233  0.004962733  0.066653784  0.010419823  0.008456776  0.016055463  0.065380083  0.075666106  0.077433304  0.832763866  0.249834108  0.45436369  0.787656031  0.847577294  0.15747466  0.277484616  0.541407016  0.174682379  0.972875031  0.670645638  0.985732627  0.62728113  0.764252042  0.704496639  0.482318157  0.574996397  0.979163739  0.697274258  0.466245387  0.201572087  0.18613163  0.904553276  0.448258264  0.400370129  0.146790355  0.359743833  0.816246026  0.767104955  0.797283008  0.416030608  0.129278508  0.389135036  0.756164818  0.232442532  0.711033283  0.492930842  0.305806121  0.988810965  0.463252095  0.723024228  0.139058989  0.766011327  0.272714159  0.455798232  0.109716535  0.537988667 | Carbohydrates  Phosphate esters  Nucleosides  Amino acids  Carbohydrates  Amino acids  Amino acids  Carbohydrates  Carbohydrates  Nucleosides  Nucleosides  Nucleosides  Nucleosides  Furanones  Organosulfonic acid  Carbohydrates  Amino acids  Nucleosides  Amino acids  Carbohydrates  Nucleosides  Keto acids  Amino acids  Nucleosides  Nucleosides  Nucleosides  Nucleosides  Nucleosides  Nucleosides  Amino acids  Nucleosides  Dicarboxylic acids  Dicarboxylic acids  Fatty acids  Hydroxy acids  Nucleosides  Amino acids  Pyridines  Amino acids  Amino acids  Nucleosides  Pyridines  Amino acids  Flavonoids  Carbohydrates  Amino acids  Carboxylic acids  Nucleosides  Nucleosides  Carbohydrates  Peptidomimetics  Amino acids  Nucleosides  Amino acids  Alcohols  Amines  Nucleosides  Amino acids  Nucleosides  Lactams  Pyridine  Indoles  Nucleosides  Amino acids  Amino acids  Carboxylic acids  Amino acids  Amino acids  Alcohols  Amino acids  Benzoic acids  Amino acids  Alcohols  Carboxylic acid  Pyrimidines  Amino acids  Amino acids  Nucleosides  Amino acids  Phenols  Nucleosides  Carbohydrates  Carboxylic acids  Fatty acids  Fatty acids  Fatty acids  Carbohydrates  Amino acids  Nucleosides  Amino acids  Nucleosides  Nucleosides  Amino acids  Nucleosides  Nucleosides  Carbohydrates  Nucleosides  Nucleosides  Phenols  Amino acids  Amino acids  Carbohydrates  Amino acids  Phenols  Fatty acids  Fatty acids  Phenylpyruvic acids  Amino acids |

^1^The metabolite mean intensities are presented as fold changes compared to unaffected tissues.

^2^The untargeted metabolomics profiling has been submitted to MetabolLights database (<https://www.ebi.ac.uk/metabolights>) under study MTBLS1493 and it is in curation.
