# Supplementary figures and images for: Muscle Metabolome Profiles in Woody Breast-(un)Affected Broilers: Effects of Quantum Blue Phytase-Enriched Diet
Source: Front Vet Sci. 2020 Aug 4;7:458. doi: 10.3389/fvets.2020.00458 (PMC7417653; doi:10.3389/fvets.2020.00458)

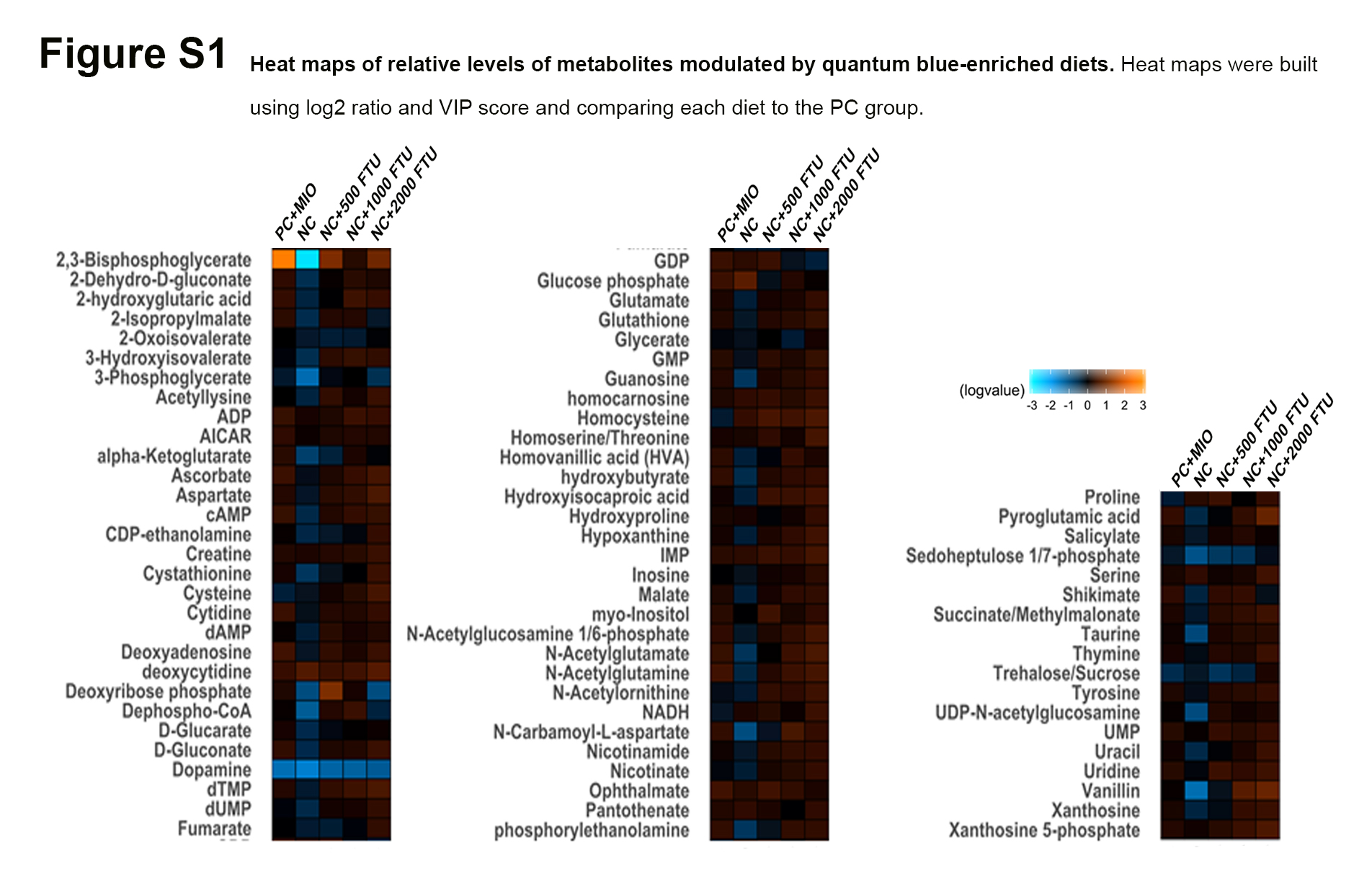

Supplement: Supplementary file 2 [file Image_1.jpg]
